# Grid technology in tissue-based diagnosis: fundamentals and potential developments

**DOI:** 10.1186/1746-1596-1-23

**Published:** 2006-08-24

**Authors:** Jürgen Görtler, Martin Berghoff, Gian Kayser, Klaus Kayser

**Affiliations:** 1 IBM DeepComputing, Brussels, Belgium; 2 Department of Neurology, University Münster, Münster, Germany; 3 Institute of Pathology, University Freiburg, Freiburg, Germany; 4 UICC-TPCC, Institute of Pathology, Charite, Berlin, Germany

## Abstract

Tissue-based diagnosis still remains the most reliable and specific diagnostic medical procedure. It is involved in all technological developments in medicine and biology and incorporates tools of quite different applications. These range from molecular genetics to image acquisition and recognition algorithms (for image analysis), or from tissue culture to electronic communication services.

Grid technology seems to possess all features to efficiently target specific constellations of an individual patient in order to obtain a detailed and accurate diagnosis in providing all relevant information and references.

Grid technology can be briefly explained by so-called nodes that are linked together and share certain communication rules in using open standards. The number of nodes can vary as well as their functionality, depending on the needs of a specific user at a given point in time. In the beginning of grid technology, the nodes were used as supercomputers in combining and enhancing the computation power. At present, at least five different Grid functions can be distinguished, that comprise 1) computation services, 2) data services, 3) application services, 4) information services, and 5) knowledge services.

The general structures and functions of a Grid are described, and their potential implementation into virtual tissue-based diagnosis is analyzed. As a result Grid technology offers a new dimension to access distributed information and knowledge and to improving the quality in tissue-based diagnosis and therefore improving the medical quality.

## Background

Tissue-based diagnosis includes all diagnosis procedures to analyze spatial configurations of biological functional units. Most frequently cells, cellular agglutinations such as vessels, nerves, glands, etc. are being investigated. Additional structures such as gene sequences, cellular movements, or membrane potentials are covered in advanced studies [1-7].

From the medical point of view it can be distinguished between several categories, namely

a) conventional or "classical histological and cytological" diagnosis,

b) "prospective" diagnosis,

c) "indicative" diagnosis, and

d) "risk-assigned" diagnosis [8,9].

The different categories of diagnosis require different technologies to be applied, and will lead to different clinical impacts as shown in <table 1>.

**Table 1:** Diagnosis categories and medical application

**Diagnosis Category**	**Medical Task**	**Examples**	**Tools**
*Conventional*	Disease classification	Lymphoma, Chronic hepatitis, Lung carcinoma	Classic microscopy, Immunohistochemistry, Molecular biology, morphometry, structure analysis
*Prospective*	Prognosis	TNM-stage, tumor proliferation rate, Apoptosis, Adhesion,	Immunohistochemistry, morphometry, structure analysis
*Indicative*	Therapeutic agents	Hormone receptors, Herceptin	Immunohistochemistry, Molecular biology, morphometry, structure analysis
*Risk-assigned*	Risk factors	BRCA1-gene	Molecular biology

The classical diagnosis is a prerequisite for any reliable treatment of chronic diseases such as cancer or chronic inflammatory lesions, and, by the way, is by far the cheapest diagnostic medical procedure [8,9]. It is also quite independent from its medical environment, i.e., the specialization of a hospital or pathology institution in contrast to the other diagnosis types.

That of prognosis-associated information requires detailed clinical information in addition to molecular pathology investigations [8,9].

The recognition of a "risk-associated disease" such as the genetic predisposition to developing breast cancer is the duty of highly specialized (molecular genetic) institutions or departments.

Therefore, institutions involved in tissue-based diagnosis should have access to a variety of sources for data, information, and knowledge, to enable working in an efficient manner. At the same time they can provide integrated and highly abstracted information of the disease and direct the necessary treatment. This central embedding of diagnostic pathology has opened new doors in medical communication.

**Figure 1:**
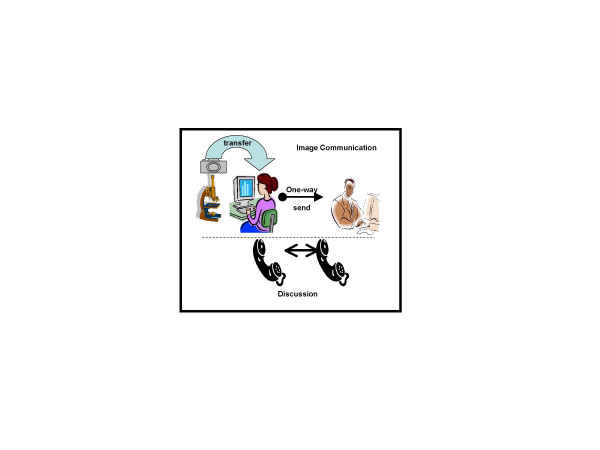
The essential tools to performing telepathology include microscope with mounted digital camera, interactive submission of clinical data and images, computerized transfer stations, and acoustic telecommunication.

It started with telepathology providing on-line and off-line procedures to electronically transfer diagnostic useful information, and continued with image analysis applications available via the Internet. The essential tools are depicted in (figure 1). On-line telepathology can be assumed as a static and asynchronous approach sending information upfront without the flexibility for the "sender" to immediately react to the reviewer's advice. User's had to "synchronize" their email communications in telephone conferences [10-23].

Another on-line technology for telepathology is the Remote Controlled Microscope. This is used by small surgical units, which do not host a surgical pathologist. The installed remote control microscopes require also "visually controlled tissue sampling and cutting tables". The systems permit intra-operative diagnosis of pathologists working with a congruent control and survey system installed in a remote pathology department or institution [24-31].

Different to these on-line telepathology systems the so-called off-line telepathology has been developed. Specific servers have been implemented to enable expert consultation, secondary advices, or to provide even a "virtual pathology institution" capability [9,10,32-41]. These systems are usually completely embedded into the Internet. Three main systems have been implemented so far, the iPATH [10,18,42] in Basel, Switzerland; the UICC-TPCC (Telepathology Consultation Center of the Union International Contre Cancer in Berlin, Germany [40,43], and the Telepathology service of the Armed Forces Institute of Pathology (AFIP), located in Bethesda, Maryland, USA [40,43-46]. These platforms allow sending information between distributed users; however, there is no interaction with communication systems or to grant access to computation facilities or specific data bases.

Another system, the Electronic Automated Measurement User System (EAMUS™, [47]) automatically measures the staining intensities and derived features of images acquired from immunohistochemically stained glass slides. It is an open system and can be accessed via the Internet [48,49].

Obviously, these systems are all build on a specific purpose and cannot interact with each other. They can be considered to be precursors of more advanced and broader designed networks meeting the characteristics of a virtual network, a Grid.

All these systems require digital images acquired from a histological glass slide that are a prerequisite to using these tools. Today, still images of limited size (SVHS, or other formats of approximately 1000 × 1000 pixels) serve for these purposes. The glass slides are still archived in the conventional manner. However, since about two years glass slide scanning technologies are available, which acquire a complete glass slide and also provide interfaces for digital archives and support advanced Internet Communication between pathologists for interactive remote consultation [48,49]. In a next step diagnostic pathology would move on from image acquisition generating "Digital Slides", into Virtual Networking, i.e. – using a Grid.

Grids are based on open standards like PACS (Picture Archiving and Communication System) for Medical Imaging and provide a simple, fast, resilient and open framework. They are designed to generate an easy to use platform for delivering intra- and interdisciplinary collaborative medicine. Images would be one core, and Health Care Systems can share pathology, cardiology, radiology and other digital images across sites.

Grid Technology enables physicians to access and use all compute and storage resources available in a virtual network. Users are granted physical freedom from the underlying technology, enabling fast remote access. Healthcare providers can leverage computing and storage resources across multiple departments and sites. By sharing resources, Grid technology will help to eliminate hardware vendor 'lock-in' via vendor agnostic architectures.

Obviously, immediate access to different diagnostic resources will improve the patients' care and physicians' diagnosis ability. Naturally, the network has to provide security and privacy to protect the patients' confidentiality.

What are the features of a Grid? Which Grids related to tissue-based diagnosis do already exist, and which specificities can be implemented in computational diagnostic pathology? Is the design of the existing telepathology services appropriate to be migrated into an advanced Grid system? Which features are promising, which ones have to be modified, or even neglected?

This article tries to give some answers from the technological and medical point of view to these questions. In addition, we want to describe the basics of Grid technology in relation to future changes in tissue-based diagnosis, which will most likely occur, in our opinion.

## Definition and description of Grid technology

**Figure 2:**
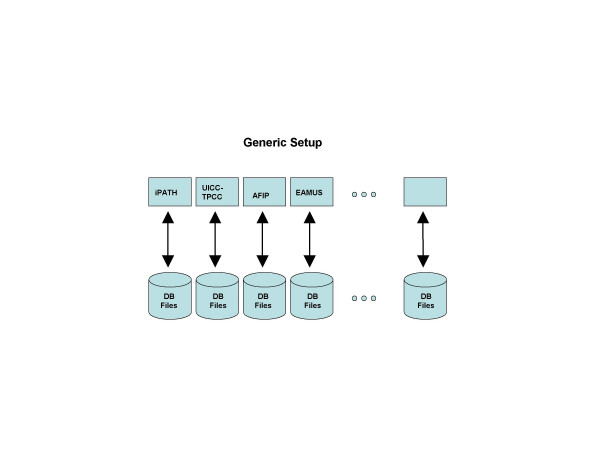
Generic setup of potential Grid services applicable for advanced expert consultation and quality assurance in tissue-based diagnosis. processing of the end user tasks.

Basically, a Grid is an Internet embedded network consisting of a broad variety of connected nodes. These nodes can be compared to servers and assure a platform of communication standards, which permit the users to concentrate solely on their individual tasks. The function of a Grid is also network computing, and can be considered to be a derivative of the development and maturation of the Internet [50]. The principle of implementation is analogue to the implementation of power supply "grids" that continuously supply households with electrical power independently where the power has been generated. A Grid uses, in place of electrical power, standardized information transfer between different nodes, for example between data sources, image servers, and highly specialized measuring systems. Similar to telephone services the user does not notice the various embedded communication pathways (e.g. cable, microwave, satellite) and computers. In addition, he is usually not informed whether he actually is connected to a computer system installed in the Far East, in Europe, or in the USA. These approaches to network computing are known as metacomputing, scalable computing, global computing, and Internet computing. Grids enable to share, select, and aggregate a broad variety of resources and devices that are geographically distributed and owned by independent organizations. The generic setup is shown in (figure 2). The main applications include large-scale computational and data intensive problems in science, engineering, and commerce.

Basically, the components of a Grid include the end users or clients, the distribution and control nodes, and the servers, anyone able to perform the requested tasks. The concept of Grid computing was primarily developed to make use of the installed compute power, which was not fully utilized (e.g. office equipment during the off-hours). The benefits are improving the execution time for a compute intensive job in linking – even geographically dispersed computers – in order to combine their computational power for this individual job. As more users might be interested using this approach all their workload has to be managed to optimize the offered capacities and services. The infrastructure of a Grid is a computer-based collaborative environment using a management software layer (Middleware). This software layer again requires computation nodes, the so-called brokers. A Grid sourced broker administers the workload, potential problems, discovers free resources, and controls the

## Grid services

A client uses a Grid to solve his specific tasks, and to receive a solution independently where and by which individual systems, called resources, it has been generated. The Grid manages the accessibility of the combined distributed resources and their services. Therefore, it is adequate to analyze the implemented types of services from the end-user's point of view. These include computational, data, application, information, and knowledge services, which can be described as follows:

*Computational services *deal with secure distributed computational resources for executing application jobs and are provided by so-called resources brokers. They serve for the set up and analysis of high energy experiments, and are also a useful tool in astrophysics. Computational services solve tasks that require high computational power, for example to solve recursive formulas. In its simplest manner, a computational task is transferred to one of the distributed supercomputers. This computer takes the job as long as it is not busy with or overloaded by other tasks. Once this happens, the task and its computational stage are transferred to another included supercomputer, etc. as long as the task is not finished. Examples of computational Grids are: NASA IPG [51], the World Wide Grid [52,53], and the NSF Tera-Grid [54,55].

*Data services *offer secure access to distributed datasets. They manage access, retrieval, storage, replication, or catalogues of individual or distributed libraries. In a more simple structure their services can be implemented by so-called links, which has been realized by several search machines. These so-called Data Grids are used in the area of high-energy physics [56] or drug design [57,58]. Another derivative is a Storage Grid as applied for Medical Imaging or data analysis in neurophysiology [59].

*Application services *manage Grid application and give access to remote software, libraries and Web services. They represent the next higher level built on computational and data services provided by the Grid. They combine the computation of specific formulas with access to prerequisite data sets. As an example, the user might be interested to viewing the shape of a new macromolecule that has some structural similarities to a known one. The application services provide the adequate formulas, and, in addition, the necessary databank of parameters etc. to fulfill this task. In tissue-based diagnosis, the EAMUS™ [9,48,49] can be considered as a simple, one node implementation of this service. A well known Grid application service is, for example, created by NetSolve [60].

*Information services *are at an advanced level of application services. They try to extract and present information provided by data of computational, information, and/or application services, and to put these into relationship. In tissue-based diagnosis, a simple implementation could be created by combining the EAMUS™ services with an existing telepathology information system such as UICC-TPCC, or iPATH. At low-level information services handle the way that information is represented, stored, accessed, shared, and maintained (Meta Data). An example of this service is the EU-sponsored Virolab Grid, a project that addresses the problem of HIV drug resistance. Its service offers the integration of biomedical information, advanced applications, patients' data, and intelligent literature access [61].

*Knowledge services *are the most advanced Grid services from the viewpoint of informatics. They are designed to supporting users in achieving their particular goals or objectives. They offer tools to improve with the way that knowledge is acquired, used, retrieved, published, or maintained. Knowledge is understood in a broad sense or as information applied to achieve a goal, solve a problem, or execute a decision. A characteristic example is data mining for automatically building a new knowledge. In tissue-based diagnosis it would be an appropriate tool in screening and evaluating virtual slides prior to be viewed by the pathologists, or to direct the clinician to providing pathologists with mandatory clinical information [8,23,62-64].

## Basic Grid structure (architecture)

**Figure 3:**
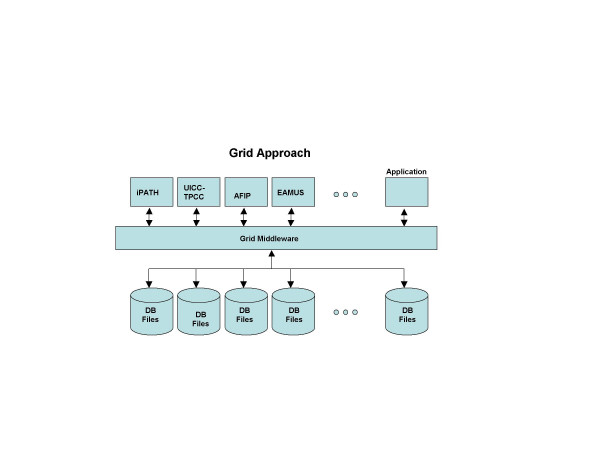
One main component of a Grid is the so-called Grid Middleware which is the backbone of the internal Grid structure.

**Figure 4:**
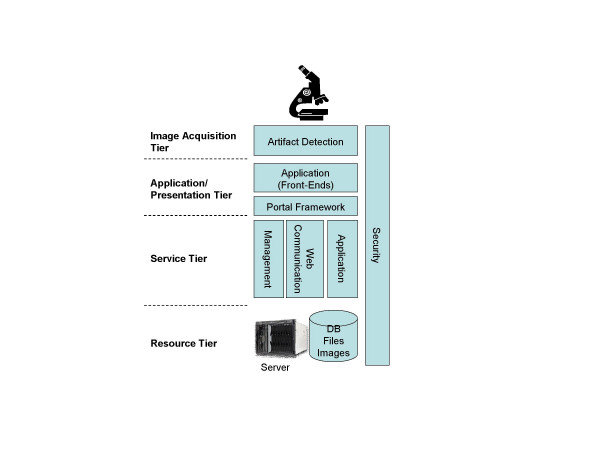
A Grid consists of a minimum of four tiers.

Grids are designed to integrate and utilize distributed resources in terms of location and functionalities. A Grid system has 1) to handle the access of the user and the presentation of the obtained results. This is done in the "presentation tier", which includes a portal framework and the application/presentation management. These (transformed and standardized) data have to be fed into the internal execution network, which is the functionality of the "service tier". These programs present the Grid middleware and provide location-independent data access, integration, transformation (standardization), and transport of data, tasks, and results as shown in (figure 3). The whole network has to be monitored. This is done by programs of the "resource tier" that check the availability of resources, their workload, dynamic status and activity of the network. In principle, a Grid is an open and dynamic communication system and requires the appropriate implementation of security services. Specific service oriented architectures (SOA) can be implemented to enable the flexibility for the Grid to adopt to changes of the workflow process, business environmental, or end user features and capabilities. The principle of a Grid structure is shown in (Figure 4). All tiers are composed of hardware and software. The *image acquisition tier *presents the scanners, digital cameras and microphones, image generation and image management SW. The control software of the microscope or scanner itself (e.g. focussing on the field of interest) can be considered being part of this tier. As image acquisition requires the glass slide preparation; this can also be assumed becoming part of image acquisition, especially if Barcode labelling and tracking is part of the implementation. The *presentation tier *includes workstations, software to handle Internet based access and data transfer, and to present the image to the pathologist. The *service tier *includes application servers, line connections, and programs to transfer and direct data streams, to interact with remote control computers, or to monitor access and response times. The *resource tier *includes the local resources in terms of management servers, information access devices such as specific measurement systems, and a broad variety of data bases and software such as libraries or specific application or execution programs. The compliance to open standards is an important aspect of any Grid component. Only these standards permit a Grid internal communication and security. They are created and internationally defined by consortia such as the Global Grid Forum (GGF), IETF, W3C and OASIS.

## Internal Grid environment

The Grid architecture consists of hardware and software that provide, control, and actualize the required functionality. It presents globally distributed resources, called the Grid fabric, as well as the Grid Middleware. Grid applications and portals to be accessible by the user can be considered the third element of the environment. Derived from these compartments four main aspects characterize a Grid in general:

### Multiple administrative domains and autonomy have to be combined, synchronized, formed to collaborative function, and supervised

Grid resources are geographically distributed and usually belong to different administrative domains and organizations. The autonomy of resource owners, their local resource management and usage policies have to be acknowledged. Their primary local function has not to be touched or even disturbed. It is quite rare that Grid resources only serve for an individual Grid; usually they provide primarily services which they have been designed to.

### Heterogeneity is a quality sign of a Grid and has to be carefully considered

Grid resources are heterogeneous in nature and encompass multiple technologies. The more can be incorporated the more attractive the Grid becomes;

### Scalability is a communication specific problem

Open communication networks are dynamic. They might grow or shrink. The physical and functional communication channels can cause remarkable delay in information transfer and speed if a communication network expands to fast. The growth of a Grid cannot be foreseen, and might raise the problem of potential performance degradation as the size of Grids increases. Consequently, applications that require a large number of resources must be adequately designed.

### Flexibility and coping with the dynamics of the resources are the main task of the Grid Middleware

The Grid Middleware provides capabilities to dynamically identify vacant and non-accessible resources and Workload Balancing ensures the efficient use of the accessible capacities.

Designing a Grid environment requires consideration of various designs to ensure the workflow and the long-term stability. For example, the definition of the information flow, supported communication protocols, file transfer technologies, networking technologies and bandwidths limitations, security and access control management etc have to be defined.

## Examples of implemented Medical Grids

The implementation of a Grid is often the joint efforts several industrial partners and scientific institutions. These include, for example, NetSolve [60], Globus [65], or Legion [66]. In diagnostic medicine, aspects of diagnostic accuracy and reliability have been in focus of Grid applications. For example, an Age-Related Eye Disease Study system for classifying age-related macular degeneration from stereoscopic color fundus photographs has been published in 2001 [67,68]. Live imaging applied for functional brain analysis by magnetic resonance technique (MRI) [69] is also undertaken with Grid technology. Grid systems to compute patients' dose, image quality and system performance in cancer screening have been described [70]. Bioinformatics Grids to be applied for analysis of genes and NDA sequences [71] are additional examples. In therapy, a new term called radio-surgery has been introduced to describe potential applications of Grid technology in surgical procedures [72]. These examples indicate that Grids have emerged as a promising technology to handle large amounts of data and compute the specific medical requirements in radiology, bioinformatics, dermatology, and neurosurgery. Especially, digital medical image processing is a promising application area for Grids that try to fill the gap between the Grid middleware and the requirements of clinical applications. A Grid system (Grid Medical Archive System, GMAS) directed to share the access, storage and retrieval of digital images obtained in radiology, cardiology, and other medical live imaging departments enables the application of the common Picture Archive and Communication System (PACS) standard and other documentation systems to access fixed-content data including medical images and documents. An extension called Grid Medical Archive Solution Entry Edition (GMAS EE) has been designed for Regional Hospitals or live imaging departments within larger hospitals to reduce the entry price point for providers while still offering all the advantages of original Grid Medical Archive Solution (GMAS) solution [73]. GMAS EE will allow Hospital Information Systems to share cardiology, radiology and other digital images across sites, and to safely store patient cases for years. This Grid is powered by IBM, and based upon Bycast StorageGRID software, a standard in grid-based fixed-content storage [74].

Recently, the European Community released a new Grid project, called ViroLab [75] or [76]. This Grid is a joined venture of the following institutions: Universiteit van Amsterdam, Institute Universitair Medisch Centrum Utrecht, Institute of Computer Science AGH, Academic Computer Centre Cyfronet, Universita Degli Studi di Brescia, Universita Cattolica del Sacro Cuore, Institute de recerca de la SIDA, Katholieke Universiteit Leuven, Eotvos Lorand Tudomanyegetem, University College London, Virology Education B.V, and Universitaet Stuttgart. Its infrastructure has been designed and built by GridwiseTech, a company specialized in Grid computing [76]. The official starting date of the project was March, 1, 2006. Virolab has been designed as virtual laboratory focusing on viral infections, especially HIV/AIDS. The Virtual Laboratory will include tools to submit data for statistical analysis, visualization, modelling and simulation. Access to patients' data and genetic information will allow clients to prognosticate the temporal virological and immunological response of viruses with complex mutation patterns to drug therapy.

## Potential Grid solution in tissue-based diagnosis

Grid-powered image storage and retrieval systems based upon Picture Archive and Communication System (PACS) applications have been developed for live imaging, neurosurgery, or dermatology. Examples have been reported in [51,72,77-80]. In contrast to these reports, implementations of Grids to be applied in tissue-based diagnosis have not been published to our knowledge. There are descriptions of systems that automatically evaluate cytology smears [81-83], or automated measure DNA content or expression of antigens [9,84,85], however, these tools can only be considered as precursors and do not meet the performance of a Grid in general, as they are designed for one analysis system with open access.

**Figure 5:**
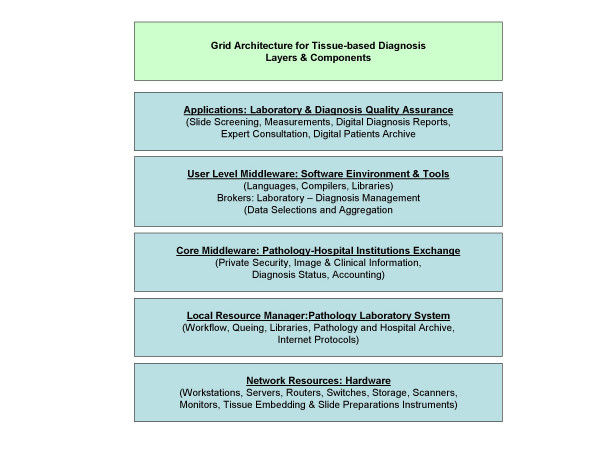
A layered Grid architecture to be applied in tissue-based diagnosis, according to [92].

It would be quite difficult to build a tissue-based diagnosis Grid that includes the performance of conventional microscopes as its application would be limited. Furthermore, it would remarkable influence the common workflow in a pathology institution. The recently technologic progress in digitalization of the whole glass slide (creation of virtual slides) will open a new era in diagnostic pathology and probably promote Grid applications in tissue-based pathology. Virtual slides are digitized images and can be easily submitted to any computational procedure [8,32,48,63,86-90]. Thus, they are contemporary an appropriate client and a useful resource in an information or even knowledge Grid. The potential architecture of such a Grid is shown in (figure 5). The provided applications include a broad range of features that cannot or only to a minor part be fulfilled even by extended certification procedures. To be mentioned have: quality assurance of scanned images, completeness check of the scanned areas, selection of diagnosis – significant areas, segmentation of objects and structures, digital structure of dictations, statistical analysis of diagnosis reports, standardized interfaces to hospital information systems, digital patients archive, embedding of an expert consultation system, and access to public libraries. Some of these resources do already exist. The EAMUS™ system [91], for example, already provides the client with image quality checks and image measurements. Its proposed extension is designed to perform an image screening to predefine the most likely diagnosis. Other resources have to be developed, especially the identification of diagnosis – significant image areas. The duty of the brokers to be installed at the users' level middleware is to administer the laboratory – surgical pathologists – secretary interactions, to manage the acute case – history relation, and to aggregate the patient's data to "a case". The internet based communication service has to regulate and control especially pathology <-> hospital, and pathology <-> research institution coupling services. It is the so-called core middle ware and has to ensure the privacy of the patient, a secure information transfer, direct access to the present status of the diagnostic procedure, and to initiate, control, and finalize the reimbursements. Finally, the Grid has to work with hardware components, which define and control the speed of the data flow, the image and connection quality, as well as the accuracy of the underlying technical procedures.

The proposed Grid realizes a virtual pathology institution. It acts simultaneously as data source, data processing, and posting (i.e., diagnosis releasing) system. The released diagnosis depends significantly on small image areas that contain the "diagnosis clue". To create a reliable Grid resource to selecting these small image compartments is probably the most difficult task of the proposed Grid. Whether this algorithm can be based upon numerical procedures or has to rely on predefined image examples still remains an open question.

In aggregate, we are convinced that Grid technology will be implemented in diagnostic surgical pathology in the near future. The process of glass slide digitalization will open the door to combine all available information resources in order to furthermore establish tissue-based diagnosis in the medical environment as it is the most reliable and even cheapest diagnostic procedure in numerous and social important diseases, such as cancer or chronic inflammatory lesions.

## References

[B1] Kayser K, Oehmann A (2004). Digital Lung Pathology.

[B2] Kayser K, Kayser G, Radziszowski D, Oehmann A (2004). New developments in digital pathology: from telepathology to virtual pathology laboratory. Stud Health Technol Inform.

[B3] Kayser K, Szymas J, Weinstein RS (2005). Telepathology and Telemedicine – Communication, Electronic Education and Publication in e-Health.

[B4] Kononen J, Bubendorf L, Kallioniemi A, Barlund M, Schraml P, Leighton S, Torhorst J, Mihatsch MJ, Sauter G, Kallioniemi OP (1998). Tissue microarrays for high-throughput molecular profiling of tumor specimens. Nat Med.

[B5] Leong FJ (2001). Practical applications of Internet resources for cost-effective telepathology practice. Pathology.

[B6] Maraki D, Yalcinkaya S, Pomjanski N, Megahed M, Boecking A, Becker J (2006). Cytologic and DNA-cytometric examination of oral lesions in lichen planus. J Oral Pathol Med.

[B7] Wikenheiser-Brokamp KA (2006). Retinoblastoma family proteins: insights gained through genetic manipulation of mice. Cell Mol Life Sci.

[B8] Kayser K, Kayser G, Gu J, Ogilvie R (2005). Virtual Microscopy and Automated Diagnosis. Virtual Microscopy and Virtual Slides in Teaching, Diagnosis and Research.

[B9] Kayser G, Radziszowski D, Bzdyl P, Sommer R, Kayser K (2006). Theory and implementation of an electronic, automated measurement system for images obtained from immunohistochemically stained slides. Anal Quant Cytol Histol.

[B10] Brauchli K, Christen H, Haroske G, Meyer W, Kunze KD, Oberholzer M (2002). Telemicroscopy by the Internet revisited. J Pathol.

[B11] Briscoe D, Adair CF, Thompson LD, Tellado MV, Buckner SB, Rosenthal DL, O'Leary TJ (2000). Telecytologic diagnosis of breast fine needle aspiration biopsies. Intraobserver concordance. Acta Cytol.

[B12] Kayser K, Szymas J, Weinstein RS (1999). Telepathology: Telecommunication, Electronic Education and Publication in Pathology.

[B13] Kayser K (2000). Telepathology in Europe. Anal Cell Pathol.

[B14] Kayser K (2002). Interdisciplinary telecommunication and expert teleconsultation in diagnostic pathology: present status and future prospects. J Telemed Telecare.

[B15] Kayser K, Szymas J, Weinstein RS (2005). Telepathology and Telemedicine Communication, Electronic Education and Publication in e-Health.

[B16] Leong FJ, Nicholson AG, McGee JO (2002). Robotic telepathology: efficacy and usability in pulmonary pathology. J Pathol.

[B17] Leong FJ, Leong AS (2004). Digital imaging in pathology theoretical and practical considerations, and applications. Pathology.

[B18] Oberholzer M, Christen H, Haroske G, Helfrich M, Oberli H, Jundt G, Stauch G, Mihatsch M, Brauchli K (2003). Modern telepathology: a distributed system with open standards. Curr Probl Dermatol.

[B19] Schwarzmann P, Binder B, Klose R (2000). Technical aspects of telepathology with emphasis on future development. Anal Cell Pathol.

[B20] Weinstein RS (1991). Telepathology comes of age in Norway. Hum Pathol.

[B21] Weinstein RS, Bloom KJ, Krupinski EA, Rozek LS (1992). Human performance studies of the video microscopy component of a dynamic telepathology system. Zentralbl Pathol.

[B22] Weinstein MH, Epstein JI (1997). Telepathology diagnosis of prostrate needle biopsies. Hum Pathol.

[B23] Weinstein RS, Descour MR, Liang C, Bhattacharyya AK, Graham AR, Davis JR, Scott KM, Richter L, Krupinski EA, Szymus J, Kayser K, Dunn BE (2001). Telepathology overview: from concept to implementation. Hum Pathol.

[B24] Battmann A, Knitza R, Janzen S, Fiedler F, Stock B, Schulz A, Knoblauch B (2000). Telemedicine: application of telepathology-remote microscopy for intraoperative diagnoses on frozen sections. Stud Health Technol Inform.

[B25] Della Mea V, Cataldi P, Pertoldi B, Beltrami CA (2000). Combining dynamic and static robotic telepathology: a report on 184 consecutive cases of frozen sections, histology and cytology. Anal Cell Pathol.

[B26] Delta Mea V, Cataldi P, Pertoldi B, Beltrami CA (1999). Dynamic robotic telepathology: a preliminary evaluation on frozen sections, histology and cytology. J Telemed Telecare.

[B27] Demichelis F, Barbareschi M, Boi S, Clemente C, Dalla Palma P, Eccher C, Forti S (2001). Robotic telepathology for intraoperative remote diagnosis using a still-imaging-based system. Am J Clin Pathol.

[B28] Kayser K, Beyer M, Blum S, Kayser G (2000). Recent developments and present status of telepathology. Anal Cell Pathol.

[B29] Oberholzer M, Fischer HR, Christen H, Gerber S, Bruhlmann M, Mihatsch MJ, Gahm T, Famos M, Winkler C, Fehr P (1995). Telepathology: frozen section diagnosis at a distance. Virchows Arch.

[B30] Singh N, Akbar N, Sowter C, Lea KG, Wells CA (2002). Telepathology in a routine clinical environment: implementation and accuracy of diagnosis by robotic microscopy in a one-stop breast clinic. J Pathol.

[B31] Wolf G, Petersen I, Dietel M (1998). Microscope remote control with an Internet browser. Anal Quant Cytol Histol.

[B32] Kayser K, Kayser G, Schrader T, Hufnagl P, ZK, DM (2003). Recent developments and future aspects of telepathology in Europe. Proceedings e_he@lth in Common Europe.

[B33] Mireskandari M, Kayser G, Hufnagl P, Schrader T, K K (2004). Teleconsultation in diagnostic pathology: experience from Iran and Germany with the use of two European telepathology servers. Journal of Telemedicine and Telecare.

[B34] Chorneyko K, Giesler R, Sabatino D, Ross C, Lobo F, Shuhaibar H, Chen V, Elavathil L, Denardi F, Ansari S, Salama S, LeBlanc V, Norman G, Sheridan B, Riddell R (2002). Telepathology for routine light microscopic and frozen section diagnosis. Am J Clin Pathol.

[B35] Szymas J, Papierz W, Danilewicz M (2000). Real-time teleneuropathology for a second opinion of neurooncological cases. Folia Neuropathol.

[B36] Szymas J, Wolf G (1999). Remote microscopy through the internet. Pol J Pathol.

[B37] Ferreira R, Moon B, Humphries J, Sussman A, Saltz J, Miller R, Demarzo A (1997). The Virtual Microscope. Proc AMIA Annu Fall Symp.

[B38] Morrison ML, McCluggage WG, Price GJ, Diamond J, Sheeran MR, Mulholland KM, Walsh MY, Montironi R, Bartels PH, Thompson D, Hamilton PW (2002). Expert system support using a Bayesian belief network for the classification of endometrial hyperplasia. J Pathol.

[B39] Schrader T, Feig T, Hufnagl P, Kayser K, Dietel M (2003). A userfriendly Telepathology Service at the Internet – The Telepathology Consultation Center of the UICC. Elec J Pathol Histol.

[B40] Dietel M, Nguyen-Dobinsky TN, Hufnagl P (2000). The UICC Telepathology Consultation Center. International Union Against Cancer. A global approach to improving consultation for pathologists in cancer diagnosis. Cancer.

[B41] Hufnagl P, Bayer G, Oberbamscheidt P, Wehrstedt K, Guski H, Hauptmann S, Dietel M (2000). Comparison of different telepathology solutions for primary frozen section diagnostic. Anal Cell Pathol.

[B42] Brauchli K, Christen H, Meyer P, Haroske G, Meyer W, Kunze KD, Otto R, Oberholzer M (2000). Telepathology: design of a modular system. Anal Cell Pathol.

[B43] Dietel M, Hufnagl P (2001). [Electronic communication in medicine]. Z Arztl Fortbild Qualitatssich.

[B44] Williams BH (1998). The AFIP center for telemedicine application – pathology for the twenty-first century. Telemed Virtual Real.

[B45] Williams BH, Mullick FG, Becker RL, Kyte RT, Noe A (1998). A national treasure goes online: the Armed Forces Institute of Pathology. MD Comput.

[B46] Williams BH, Mullick FG, Butler DR, Herring RF, O'Leary JT (2001). Clinical evaluation of an international static image-based telepathology service. Hum Pathol.

[B47] http://www.eamus.de.

[B48] Kayser K, Radziszowski D, Bzdyl P, Sommer R, Kayser G (2006). Digitized pathology: theory and experiences in automated tissue-based virtual diagnosis. Rom J Morphol Embryol.

[B49] Kayser K, Radziszowski D, Bzdyl P, Sommer R, Kayser G (2006). Towards an automated virtual slide screening: theoretical considerations and practical experiences of automated tissue-based virtual diagnosis to be implemented in the Internet. Diagn Pathol.

[B50] Oliveira IC, Oliveira JL, Sanchez JP, Lopez-Alonso V, Martin-Sanchez F, Maojo V, Sousa Pereira A (2005). Grid requirements for the integration of biomedical information resources for health applications. Methods Inf Med.

[B51] Johnston W, Gannon D, Nitzberg B (1999). Grids as production computing environments: The engineering aspects of NASA's information power Grid. Eighth IEEE International Symposium on High Performance Distributed Computing, Redondo Beach, CA, August 1999.

[B52] http://www.buyya.com/ecogrid/wwg/.

[B53] Buyya R (2006). The World-Wide Grid. http://www.buyya.com/ecogrid/wwg/.

[B54] NSF, T-G (2006). NSF Tera-Grid. http://www.teraGrid.org/.

[B55] http://www.teraGrid.org/.

[B56] Hoschek W, Jaen-Martinez J, Samar A, Stockinger H, Stockinger K (2000). Data management in an international data Grid project. Proceedings of the 1st IEEE/ACM International Workshop on Grid Computing (Grid'2000), Bangalore, India, 17–20 December 2000.

[B57] Buyya R (2001). The Virtual Laboratory Project: Molecular modeling for drug design on Grid. IEEE Distributed Systems Online.

[B58] http://www.buyya.com/vlab/.

[B59] Nakagawa S, Kosaka T, Date S, Shimojo S, Tonoike M (2004). A grid computing infrastructure for MEG data analysis. Neurol Clin Neurophysiol, 2004.

[B60] Casanova H, Dongarra J (1997). NetSolve: A network server for solving computational science problems. International Journal of Supercomputing Applications and High Performance Computing.

[B61] http://www.gridwisetech.com/virolab.

[B62] Weinstein RS (2003). The education of professionals. Hum Pathol.

[B63] Weinstein RS, Descour MR, Liang C, Barker G, Scott KM, Richter L, Krupinski EA, Bhattacharyya AK, Davis JR, Graham AR, Rennels M, Russum WC, Goodall JF, Zhou P, Olszak AG, Williams BH, Wyant JC, Bartels PH (2004). An array microscope for ultrarapid virtual slide processing and telepathology. Design, fabrication, and validation study. Hum Pathol.

[B64] Weinstein RS, Decour MR, Liang C, Richter L, Russum WC, Goodall JF, Zhou P, Olzak AG, Bartels PH, Gu J, Ogilvie R (2005). Reinventation of light microscopy: array microscopy and ultrarapidly scanned virtual slides for diagnostic pathology and education. Virtual Microscopy and Virtual slides in Teaching, Diagnosis, and Research.

[B65] Foster I, Kesselman C (1997). Globus: A metacomputing infrastructure toolkit. International Journal of Supercomputer Applications.

[B66] Grimshaw A, Wulf W (1997). The Legion vision of a worldwide virtual computer. Communications of the ACM.

[B67] Johnson CA, Cioffi GA, Van Buskirk EM (1999). Frequency doubling technology perimetry using a 24 – 2 stimulus presentation pattern. Optom Vis Sci.

[B68] (2001). The Age-Related Eye Disease Study system for classifying age-related macular degeneration from stereoscopic color fundus photographs the Age-Related Eye Disease Study Report Number 6. Am J Ophthalmol.

[B69] Bagarinao E, Matsuo K, Tanaka Y, Sarmenta LF, Nakai T (2005). Enabling on-demand real-time functional MRI analysis using grid technology. Methods Inf Med.

[B70] Brandan ME, Ruiz-Trejo C, Verdejo-Silva M, Guevara M, Lozano-Zalce  H, Madero-Preciado L, Martin J, Noel-Etienne LM, Ramirez-Arias JL, Soto J, Villasenor Y (2004). Evaluation of equipment performance, patient dose, imaging quality, and diagnostic coincidence in five Mexico City mammography services. Arch Med Res.

[B71] Carvalho PC, Gloria RV, de Miranda AB, Degrave WM (2005). Squid – a simple bioinformatics grid. BMC Bioinformatics.

[B72] Fenner JW, Mehrem RA, Ganesan V, Riley S, Middleton SE, Potter K, Walton L (2005). Radiosurgery planning supported by the GEMSS grid. Stud Health Technol Inform.

[B73] http://www-03.ibm.com/industries/healthcare/.

[B74] http://www.bycast.com.

[B75] http://www.virolab.org:8080/virolab/.

[B76] http://www.gridwisetech.com/virolab.

[B77] Germain C, Breton V, Clarysse P, Gaudeau Y, Glatard T, Jeannot E, Legre Y, Loomis C, Magnin I, Montagnat J, Moureaux JM, Osorio A, Pennec X, Texier R (2005). Grid-enabling medical image analysis. J Clin Monit Comput.

[B78] Akiyama T, Teranishi Y, Nozaki K, Kato S, Shimojo S, Peltier ST, Lin A, Molina T, Yang G, Lee D, Ellisman M, Naito S, Koike A, Matsumoto S, Yoshida K, Mori H (2005). Scientific Grid activities and PKI deployment in the Cybermedia Center, Osaka University. J Clin Monit Comput.

[B79] Balogh AA, Preul MC, Laszlo K, Schornak M, Hickman M, Deshmukh P, Spetzler RF (2006). Multilayer image grid reconstruction technology: four-dimensional interactive image reconstruction of microsurgical neuroanatomic dissections. Neurosurgery.

[B80] Liu BJ, Zhou MZ, Documet J (2005). Utilizing data grid architecture for the backup and recovery of clinical image data. Comput Med Imaging Graph.

[B81] Husain OA, Watts KC (1987). Preparatory methods for DNA hydrolysis, cytochemistry, immunocytochemistry and ploidy analysis. Their application to automated and routine diagnostic cytopathology. Anal Quant Cytol Histol.

[B82] Zahed L, Murer-Orlando M, Bobrow M (1989). The application of automated metaphase scanning to direct preparations of chorionic villi. Prenat Diagn.

[B83] Husain OA, Watts KC, Lorriman F, Butler B, Tucker J, Carothers A, Eason P, Farrow S, Rutovitz D, Stark M (1993). Semi-automated cervical smear pre-screening systems: an evaluation of the Cytoscan-110. Anal Cell Pathol.

[B84] Haroske G, Meyer W, Oberholzer M, Bocking A, Kunze KD (2000). Competence on demand in DNA image cytometry. Pathol Res Pract.

[B85] Haroske G, Meyer W, Kunze D, Boeking A (1998). Quality control measures for dna image cytometry in a telepathology network. Adv Clin Path.

[B86] Fujita K, Crowley RS (2003). The Virtual Slide Set – a curriculum development system for digital microscopy. AMIA Annu Symp Proc.

[B87] Lundin M, Lundin J, Helin H, Isola J (2004). A digital atlas of breast histopathology: an application of web based virtual microscopy. J Clin Pathol.

[B88] Gu J, Ogilvie R, edts (2005). Virtual Microscopy and Virtual Slides in Teaching, Diagnosis, and Research.

[B89] Dee FR, Heidger P, Gu J, Ogilvie RW (2005). Virtual Slides for teaching Histology and Pathology. Virtual Microscopy and Virtual Slides in Teaching, Diagnosis, and Research.

[B90] Burthem J, Brereton M, Ardern J, Hickman L, Seal L, Serrant A, Hutchinson CV, Wells E, McTaggart P, De la Salle B, Parker-Williams J, Hyde K (2005). The use of digital 'virtual slides' in the quality assessment of hematological morphology results of a pilot exercise involving UK NEQAS (H) participants. British Journal of Haematology.

[B91] http://www.pathology-online.org/.

[B92] Kayser K, Molnar B, Weinstein RS (2006). Virtual Microscopy – Fundamentals – Applications – Perspectives of Electronic Tissue-based Diagnosis.

